# A Misuse of IQ Scores: Using the Dual Discrepancy/Consistency Model for Identifying Specific Learning Disabilities

**DOI:** 10.3390/jintelligence6030036

**Published:** 2018-08-01

**Authors:** A. Alexander Beaujean, Nicholas F. Benson, Ryan J. McGill, Stefan C. Dombrowski

**Affiliations:** 1 Psychology & Neuroscience Department, Baylor University, Waco, TX 76798, USA; 2 Educational Psychology Department, Baylor University, Waco, TX 76798, USA; Nicholas_Benson@baylor.edu; 3 School of Education, College of William & Mary, Williamsburg, VA 23187, USA; rmcgill@wm.edu; 4 Department of Graduate Education, Leadership and Counseling, Rider University, Lawrenceville, NJ 08648, USA; sdombrowski@rider.edu

**Keywords:** dual discrepancy/consistency, pattern of strengths and weaknesses, cognitive profile analysis, specific learning disability

## Abstract

The purpose of this article is to describe the origins of patterns of strengths and weaknesses (PSW) methods for identifying specific learning disabilities (SLD) and to provide a comprehensive review of the assumptions and evidence supporting the most commonly-used PSW method in the United States: Dual Discrepancy/Consistency (DD/C). Given their use in determining whether students have access to special education and related services, it is important that any method used to identify SLD have supporting evidence. A review of the DD/C evidence indicates it cannot currently be classified as an evidence-based method for identifying individuals with a SLD. We show that the DD/C method is unsound for three major reasons: (a) it requires test scores have properties that they fundamentally lack, (b) lack of experimental utility evidence supporting its use, and (c) evidence supporting the inability of the method to identify SLD accurately.

## 1. Introduction

The study of intelligence and education have long been intertwined.^1^ The first widely used IQ test, created by Alfred Binet [1], was developed to be used within elementary schools to identify students who had an intellectual disability. After Goddard brought Binet’s instrument to the United States (US), the popularity of intelligence research and clinical use saw a remarkable growth [2].

The popularity of intelligence assessment grew even more after David Weschler [3] published his Bellevue instrument. The novelty of the Wechsler–Bellevue was that all the subtests were scaled so that they had the same mean and standard deviation. Not only did this allow clinicians to use IQ tests to examine delays in intellectual development, but it also allowed clinicians to use them to diagnose other forms of psychopathology (e.g., schizophrenia, bipolar disorder) [4]. Such diagnoses were made predicated on the idea that intelligence is best understood as a group of multiple, functionally independent attributes whose development is relatively uniform. Thus, if an some intelligence attributes were less developed than others for a given individual (i.e., there were strengths and weaknesses within the individual), it was likely due to some “unorganized or disordered personality functioning in the cognitive, affective, and instinctual areas of behavior” [5] (p. 178). In order to assess this “unorganized functioning”, clinicians had to engaged in *cognitive profile analysis*: examining intra-individual differences in IQ test scores.^2^

Examining cognitive profiles became popular among clinical psychologists, but there were individuals who noted that there was little experimental evidence to support their interpretation [6].^3^ In fact, much research in the mid-twentieth century was devoted to examining the utility of cognitive profiles to diagnose different forms of psychopathology. Much of this research failed to support the use of cognitive profiles [8,9].

Despite the lack of experimental evidence supporting the interpretation of cognitive profiles, it remained popular with clinicians. To some extent, the lack of evidence was acknowledged. Instead of limiting the practice of cognitive profile analysis, however, it was re-framed. First, instead of score variability being due to an “unorganized or disordered personality”, it was more likely due to some problem with cognition or learning. Second, interpreting cognitive profiles could not be done by just any psychologist. Instead, it was a skill that could only be acquired through intensive training with clinicians who were well-versed in the method—i.e., it was an art instead of a science [10].

In the mid-twentieth century, the popularity of the cognitive profile analysis began to manifest itself in special education [11]. School/educational psychologists and special education professionals began using scores from IQ tests and related instruments to aid in the diagnosis of learning difficulties and design specific interventions. From the late 1960s through the mid 1990s, a panoply of cognitive profile approaches—most of which were based on Wechsler instrument subtests—were developed and presented to practitioners as potentially useful for identifying individuals with learning difficulties [12,13]. As with their use in clinical psychology, experimental studies showed that cognitive profiles had limited diagnostic accuracy in identifying individuals with learning difficulties [14,15,16].

Despite the dearth of experiments supporting the practice, the idea that learning difficulties could be diagnosed, and subsequent interventions developed, through examining cognitive profiles has continued to remain popular among clinicians and educators. In fact, a whole cottage industry has developed around the use of cognitive profiles. Individuals interested in this approach to IQ test score interpretation can choose from a variety of workshops, books, software, and even online certificate programs in order to hone their skills in finding and interpreting various cognitive profiles.

While the practice of using cognitive profiles to identify learning problems has been called multiple names throughout its history, it currently goes by the umbrella term of *patterns of strengths and weaknesses* (PSW). The purposes of this critique are to describe the origins of PSW methods as they relate to specific learning disability (SLD) identification and to provide a comprehensive review of the assumptions and evidence for one particular PSW method: Dual Discrepancy/Consistency [17]. We address it specifically because research suggests it is the most common PSW method used in the US for SLD identification [18]. Regardless of popularity, it is important that any method used to identify SLD is evidence-based given its use to determine whether or not students have access to special education and related services.

### 1.1. Identification: Unexpected Underachievement

*Specific learning disability* is an umbrella term that describes a condition where individuals display low achievement that cannot be explained by other disorders or environmental factors [19]. Although the study of SLDs started in the nineteenth century, it largely became introduced into the US educational system in the 1960s [20]. By 1969, a federal definition of SLD was developed and included as part of the Children with Specific Learning Disabilities Act, which was later consolidated into the Education of the Handicapped Act (Public Law 91-230) in 1970 [21].

The 1969 definition remains largely unchanged as of the most recent authorization of the Individuals with Disabilities Education Act (IDEA; Public Law 108-446). The federal definition of SLD requires the existence of a “disorder in one or more of the basic psychological processes” (34 CFR § 300.8.c.10). The major problem with this definition is that it is not technical, thus determining if a student had a SLD was a very subjective process.

In 1976, the US Office of Education tried to improve the situation by adding an operational diagnostic criterion of a “severe discrepancy between achievement and intellectual ability in one or more of several areas” [22] (p. 52406). The diagnostic criterion is curious because it did not address basic psychological processes—one of the key components the original definition [23]—or how one determines if there is a disorder in such a process. Instead, the focus on basic psychological processes is replaced with a discrepancy method for identifying SLD. Subsequently, the assessment of SLD—at least by school systems—required demonstration that an individual demonstrated “unexpected underachievement” [24,25]. Unexpected underachievement was not directly assessed, however; it was inferred when individuals did not acquire academic skills at an expected rate in conjunction with an absence of exclusionary factors (i.e., other reasons that could cause low academic achievement, such as visual/hearing/motor impairments, intellectual disability, emotional/behavioral difficulties, limited English proficiency, or lack of adequate instruction).

The nebulous nature of the operationalization (i.e., severe discrepancy) was purposeful—there was little consensus about the characteristics of SLD outside of “unexpected underachievement” [26]. Thus, it was left up to each state to determine how to measure the discrepancy. Most states adopted an approach that examined the difference between a standardized norm-referenced IQ test score and a standardized norm-referenced academic achievement test score. The idea undergirding this IQ-achievement discrepancy (IAD) approach is that IQ test scores represent individuals’ capacity for learning while achievement test scores represent individuals’ actual level of learning. Thus, if the difference was sufficiently large, this was taken as evidence that an individual may have a SLD.

The situation changed in 2004 when the US Congress enacted new guidelines for diagnosing the impairment with amendments to the IDEA. Because the scientific literature noted many problems with the IAD method [27,28], it was dropped as being required. Instead of taking a clear position on how SLD should be identified, however, Congress basically left the decision up to the individual states. The regulations specifically allow for the IAD approach, response-to-intervention (RtI), as well as “other alternative research-based procedures” (34 CFR § 300.307.a.3). The alternative research-based procedures option was included, and purposefully left undefined, to give states the flexibility to allow methods that could not be categorized as either IAD or response-to-intervention (RtI) [29] (p. 46648).

To date, there have largely been two common frameworks for the alternative research-based procedures used to identify SLD: (a) instructional, and (b) discrepancy [30]. Instructional frameworks focus on low achievement that occurs in the absence of exclusionary factors. Some examples are the “integrated” or “hybrid” methods [31,32]. Since this approach is not the focus of this article, we do not discuss it further. Instead, we turn our attention to discrepancy frameworks.

### 1.2. Pattern of Strengths and Weaknesses

Discrepancy frameworks assume that SLDs are marked by academic difficulties that are unexpected based on the presence of otherwise normal (or better-than-normal) intellectual functioning and the absence of exclusionary factors. Under the “alternative research-based procedures” framework, the most popular method is the investigation of pattern of strengths and weaknesses. The term *pattern of strengths and weaknesses* is taken from the language in IDEA used to describe SLD identification criteria (34 CFR. § 300.309). Specifically,

The child exhibits a pattern of strengths and weaknesses in performance, achievement, or both, relative to age, state-approved grade-level standards, or intellectual development, which is determined by the group to be relevant to the identification of a specific learning disability, using appropriate assessments, consistent with §§ 300.304 and 300.305. (34 CFR. §$\tilde{3}$00.309.a.2.ii).

From one perspective, this method is different from IAD. IAD methods use a single IQ test score as a marker for general learning capacity, whereas PSW methods require the existence of an unevenness in the development of intelligence attributes as manifested by patterns of IQ test scores. Individuals with SLD are those with strengths in many intelligence attributes but weaknesses in others that are thought to lead to underachievement in one or more academic areas. From another perceptive, PSW methods are just extension of IAD. Like IAD, PSW methods require a discrepancy between scores from IQ test scores and norm-referenced achievement tests. Instead of a single discrepancy, however, PSW methods require the presence of a certain profile of IQ and academic achievement scores.

To date, there have been four major operationalizations of PSW: (a) concordance/discordance [33]; (b) discrepancy/consistency [34]; (c) core-selective [35]; and (d) dual discrepancy/consistency [17]. There are many similarities among the methods, but they also differ in important ways [36,37]. We focus on the dual discrepancy/consistency method because it is the most common one currently used in the US [18].

## 2. Dual Discrepancy/Consistency

The dual discrepancy/consistency (DD/C) method is an outgrowth of cross-battery assessment (XBA) [38], which is a particular approach to intelligence assessment. XBA uses the Cattell–Horn–Carroll (CHC) [39] taxonomy of intelligence test scores as a foundation for determining intelligence attributes that should be examined in a typical intellectual assessment. Of particular note, the CHC taxonomy was developed independently of XBA. Thus, XBA would not exist without the CHC taxonomy, but the CHC taxonomy could exist without XBA.

In XBA, clinicians administer subtests from standardized nationally-normed instruments assessing intelligence attributes the CHC taxonomy classifies as broad abilities. The seven broad abilities important in XBA are: (a) Fluid Reasoning (*Gf*), (b) Comprehension-Knowledge (*Gc*), (c) Visual Processing (*Gv*), (d) Short-Term Memory (*Gwm*), (e) Auditory Processing (*Ga*), (f) Long-Term Storage and Retrieval (*Glr*), and (g) Processing Speed (*Gs*).

Clinicians can choose from a variety of IQ tests that have subtests measuring the different broad ability areas. Thus, two clinicians examining the same student using XBA may arrive at their conclusions using completely different sets of IQ tests and subtests. Currently, there is only one IQ test that assesses all of the broad abilities: Woodcock–Johnson IV [40]. Clinicians not using this instrument can obtain complete CHC-based profiles only by administering and combining subtest scores from a battery of different IQ tests—hence the term cross-battery assessment.

Flanagan et al. [41] first developed the idea of using XBA to identify a SLD. The method has gone by multiple names but is currently called DD/C [38].^4^ Its name comes from criteria required to identify a SLD: (a) low academic achievement test scores in at least one area along with low IQ test scores representing at least one broad ability (the dual discrepancy), (b) the low IQ test scores have to be related to the low academic achievement scores (the consistency), and (c) all other IQ test scores are average or higher.^5^

The DD/C method of SLD identification includes five levels of evaluation. The levels are hierarchical in that individuals have to meet the criteria at a lower level to move on to a higher level. We only describe Levels 1–4 because these levels are the crux of the DD/C method. Level 5 involves the multidisciplinary team determination of whether special education and related services are necessary, which is required for identification of all disabilities in US schools.

### 2.1. Level 1. Weaknesses or Deficits in One or More Areas of Academic Achievement

Level 1 involves a comprehensive assessment of academic achievement and requires the use of standardized nationally-normed achievement tests that provide norm-referenced scores. While Flanagan et al. [38] suggested that data on academic performance should come from multiple sources, only data from standardized achievement tests are sufficient for SLD identification.

To move to Level 2, a student must have a norm-referenced academic achievement score that is a weakness or normative weakness/deficit. A *weakness* is defined by a norm-referenced score that falls between 0.73 and 1.00 standard deviations (SDs) below the average performance, whereas a *normative weakness/deficit* is defined by a norm-referenced score that is >1.00 SD below the average performance.^6^

### 2.2. Level 2. Exclusionary Factors

Level 2 involves evaluating whether any documented weaknesses or deficits found at Level 1 are primarily the result of exclusionary factors. To move to Level 3, exclusionary factors have to be evaluated and eliminated as possible primary explanations for the poor academic performance observed in Level 1.

### 2.3. Level 3. Weaknesses or Deficits in One or More Intelligence Attribute

Level 3 involves the assessment of CHC-based abilities using standardized nationally-normed IQ tests that provide norm-referenced scores. Of particular interest are the broad abilities thought to be related to the academic weaknesses or deficits found in Level 1.

A particularly salient aspect of the (DD/C) operational definition of SLD is that a weakness or deficit in one or more cognitive abilities or processes underlies difficulties in academic performance and skill development. Because research demonstrates that the relationship between the cognitive dysfunction and the manifest learning problems are causal in nature, data analysis at this level should seek to ensure that identified weaknesses or deficits on cognitive and neuropsychology tests bear an empirical relationship to those weaknesses or deficits on achievement tests identified previously.

[38] (pp. 252–253)

For example, according to Flanagan et al. [17,38], the intelligence attributes important to assess a SLD in reading are provided in the two CHC columns of Table 1.^7^

In assessing the broad abilities, “a minimum of two qualitatively different indicators per CHC broad ability is *recommended* in the XBA approach for practical reasons (viz., time-efficient assessment)” [38] (p. 37, emphasis added). Note, it is not required, only recommended. To move to Level 4, there must be one or more weaknesses or deficits in CHC abilities.

### 2.4. Level 4. Pattern of Strengths and Weaknesses Meeting the Dual Discrepancy/Consistency Criteria

Level 4 involves analysis of the norm-referenced scores gathered at Levels 1 and 3 to determine whether the student displays the PSW required by the DD/C model within an otherwise normal IQ test score profile. Specifically, the decision to be made at this level is

whether the pattern of results is marked by an empirical or ecologically valid relationship between the identified cognitive and academic weaknesses, whether the individual displays generally average ability to think and reason, whether the individual’s learning difficulty is domain specific, and whether the individual’s underachievement is unexpected.

[38] (p. 254)

The analyses required for Level 4 *have* to be completed using the software developed by Flanagan and colleagues [17] because “specific formulae and regression equations are necessary” to make the determination about whether the IQ and achievement scores meet the DD/C criteria (p. 357). This software was originally included as part of a book [38], but now needs to be purchased separately as the *Cross-Battery Assessment Software System* (X-BASS) [45].

Individuals can be identified as having a SLD when the following criteria are met:

1. The composite general ability score (*g-Value*) is in the average range or better. The *g-Value* is calculated using a proprietary algorithm in the X-BASS program.
2. ≥1 norm-referenced IQ test score representing a CHC broad ability shows a normative weakness.
3. There is a “statistically significant” and “rare” difference between the Facilitating Cognitive Composite (FCC) and any IQ test scores that demonstrated weaknesses (i.e., intelligence attribute weaknesses).^8^ The FCC is another proprietary composite score calculated by the X-BASS program.
4. ≥1 norm-referenced score on an academic achievement test shows a normative weakness.
5. There is a “statistically significant” and “rare” difference between the FCC and any achievement test score that demonstrated weaknesses.
6. The academic weaknesses are consistent with the weaknesses in intelligence attributes (“below-average cognitive aptitude-achievement consistency”).

## 3. Research on the Dual Discrepancy/Consistency Method

### 3.1. Specific Learning Disability Research Desiderata

When examining SLD identification methods, it is not sufficient to show that IQ test scores are related to academic achievement test scores, nor is it sufficient to show that there are statistically significant mean differences between IQ test scores across SLD groups. Showing that two groups’ mean test scores significantly differ is not difficult to find; it just requires having a large enough sample size [46]. Such analyses shed little light on the question that gave rise to the study in the first place: how useful the test scores are in differentiating between individuals with and without a SLD [47].

To answer the question about whether test scores can differentiate between SLD and non-SLD groups requires evidence related to clinical or diagnostic utility [48]. *Diagnostic utility* is how well some procedure (e.g., score profile patterns) can improve the accuracy of diagnostic decision-making [49]. Applied to DD/C, examining diagnostic utility requires determining the accuracy of the DD/C criteria in identifying SLD. This means being able to reject those individuals who do not have SLD as well as identify those who do have a SLD. At a minimum, it requires data shown in Table 2 [50].^9^

When examining utility information, it is important to examine the base rate, specificity, and sensitivity (formulae provided in the Appendix.) The *base rate* (or prevalence) is the proportion of individuals with SLD. In the US, the SLD base rate is approximately 0.10 [52], meaning that if a clinician never identified anyone as having a SLD he/she would be correct approximately 90% of the time.^10^

*Specificity* is the proportion of individuals without SLD who also do not meet the DD/C criteria. *Sensitivity* is the proportion of individuals with SLD who also meet the DD/C criteria. Related to specificity and sensitivity are predictive values. Whereas specificity and sensitivity are relatively robust against variations in the base rate, predictive values incorporate the base rate into their calculations. The *negative predictive value* (NPV) is the probability that an individual predicted to not have SLD via DD/C actually does not have SLD. Since SLD is relatively rare in the general population, NPV will typically be large irrespective of the diagnostic method’s accuracy. The *positive predictive value* (PPV) is the probability that an individual predicted to have SLD via DD/C actually has SLD.

### 3.2. Arguments Used to Support the Dual Discrepancy/Consistency Method

There are many articles and chapters written about XBA in general, and the DD/C method in particular. Since many of the same cadre of individuals are involved in both XBA and DD/C, they cite themselves often, e.g., [17,38,54]. This makes for large reference sections that can look impressive to the outside observer. Reviewers of this the literature have noted problems, however, and have questioned the strength of the evidence provided [55,56].

While it is not uncommon for researchers to investigate their own methods, over-reliance on these publications can be problematic. First, the majority of the publications used to support DD/C are authored or edited by the DD/C developers, which can give the appearance of confirmation bias. Second, the DD/C developers have only produced publications that are supportive of DD/C, which can give the appearance of allegiance effects [57]. Third, the DD/C developers have a financial conflict of interest (CoI)—i.e., they financially profit when others choose to adopt the method—which could potentially cause bias in the decision-making process, even if the CoIs are disclosed [58].^11^

Support for the DD/C method largely comes in five types: (a) anecdotes, (b) reliance on CHC, (c) appeal to legal permissiveness, (d) correlations thought to imply causality, and (e) studies that answer the wrong question. Reliance on these as evidence for the utility of DD/C, however, is logically flawed [59]. We describe each of these types of support as well as the logical flaws accompanying them.

#### 3.2.1. Anecdotes

The first, and most common, type of support is commentaries about why the DD/C *should* work. These are often presented in books or book chapters in books from which at least one of primary DD/C developers is an editor, e.g., [56,60]. These pieces are often accompanied by select anecdotes as a demonstration of why it works and are then cited within the DD/C literature as evidence for utility of the method.

Case studies can be very useful when well designed and systematically implemented [61]. For example, they can aid in developing hypotheses or serve as a method of disconfirming general propositions [62]. Providing isolated anecdotes, however, without any discussion of pertinent design features (e.g., how cases were selected)—especially when the individuals providing the case studies have apparent CoIs—does not imbue trust in the credibility of the researchers’ procedures. Thus, readers are left wondering what mechanisms were put in place to protect against the authors seeming to find what they had initially set out to find.

While commentaries and anecdotes can be useful in a scientific field, they cannot replace experiments. Moreover, they provide little evidence about utility—especially when pertinent design features are not described and mechanisms are not put in place to protect against authors only selecting cases that confirm their initial hypotheses. Thus, the assumption that commentaries provide support DD/C is an anecdotal fallacy.

#### 3.2.2. Reliance on Cattell–Horn–Carroll

The second type of support is citations to research supporting the CHC taxonomy of intelligence attributes, e.g., [54,63]. Assuming the CHC taxonomy is valid, it would only provide a useful method for classifying IQ and achievement tests. It would provide no information about the validity of using this information to create a method for conducting a intelligence assessment (e.g., XBA), much less evidence about the utility of assessing certain areas in identifying a SLD (e.g., DD/C). The assumption that research supporting CHC automatically supports DD/C (or even XBA) is a fallacy of composition.

#### 3.2.3. Appeal to Legal Permissiveness

The third type of support is citations to federal statutory requirements, e.g., [64,65]. Specifically, DD/C supporters cite the “pattern of strengths and weaknesses” phrase in the definition of SLD in IDEA (34 CFR § 300.309.a.2.ii) and the “other alternative research-based procedures” option in the 2006 IDEA regulations (34 CFR § 300.307.a.3).

These arguments are misplaced and legally flawed [66]. Using the DD/C criteria requires assessing multiple intelligence attributes, but this was never the intention of the legislation. This misunderstanding is shown most clearly in the US Department of Education’s commentary on IDEA regulations.

The Department [of Education] does not believe that an assessment of psychological or cognitive processing should be required in determining whether a child has an SLD. There is no current evidence that such assessments are necessary or sufficient for identifying SLD. Furthermore, in many cases, these assessments have not been used to make appropriate intervention decisions. However, § 300.309(a)(2)(ii) permits, but does not require, consideration of a pattern of strengths or weaknesses, or both, relative to intellectual development, if the evaluation group considers that information relevant to an identification of SLD. *In many cases, though, assessments of cognitive processes simply add to the testing burden and do not contribute to interventions* (emphasis added).

[29] (p. 46651)

Consequently, the assumption that DD/C is valid because it is not legally prohibited makes it a form of the appeal to nature fallacy.

#### 3.2.4. Correlations Thought to Imply Causality

A fourth type of support provided for DD/C is the correlation between IQ test scores and standardized academic achievement scores. This evidence is then used to infer that the former causes disabilities in the the latter:

A particularly salient aspect of the [DD/C] operational definition of SLD is that a weakness or deficit in one or more cognitive abilities or processes underlies difficulties in academic performance and skill development. Because research demonstrates that the relationship between the cognitive dysfunction and the manifest learning problems are *causal* in nature …data analysis at this level [Level 3] should seek to ensure that identified weaknesses or deficits on cognitive and neuropsychology tests bear an empirical relationship to those weaknesses or deficits on achievement tests identified previously.

[38] (pp. 252–253, emphasis added)

There is a large corpus of literature showing that IQ test scores are correlated with academic achievement test scores e.g., [67]. This is entirely separate from demonstrating that either (a) some intelligence attribute has a causal relation to academic deficits, or (b) IQ test scores provide any added information for SLD identification over and above achievement test scores. The major problem with going from IQ and achievement test scores are correlated to specific intelligence attributes are causing academic performance difficulties is threefold.

First, most IQ test scores represent impure mixtures—meaning they are assessing a collection of different abilities, not a pure measure of any specific attribute [68]. Thus, the reason for the correlation could be that some of the same skills required for completing academic items are also required for IQ tests. For example, the skills assessed by reading tests are some of the same skills required for many IQ tests (see Table 1). Thus, the distinction between when items assess an intelligence attribute (via placement on an IQ test) versus when they assess some aspect of academic achievement (via placement on an academic achievement test) is arbitrary. Showing that scores on the two instruments correlate would not mean that the former causes the latter—only that they require the same set of skills to complete.

Second, the “causal” direction between the intelligence and academic achievement attributes is not always clear. Instead of Intelligence → Achievement (i.e., intelligence causes achievement), it could be that Intelligence ← Third Variable →Achievement (i.e., the relation results from a third attribute that acts a common cause of both). Or perhaps Intelligence ⇆ Achievement (i.e., the relation is reciprocal/cyclical); that is, intelligence and academic achievement attributes could be working together, each “causing” each other.

For example, Watkins et al. [69] examined intelligence attributes (*Gc* and a hybrid of *Gf* and *Gv*) and academic achievement attributes (reading and math abilities) in students referred for special education. Every student in their sample had data at two time points, with time 1 (T1) being approximately three years from time 2 (T2). Watkins et al. fit a series of cross-lagged models to examine the relations across time. In their “best” model (M2), *Gc*, *Gf/Gv*, reading, and math at T1 were specified to be direct causes of academic achievement at T2. While *Gc* and *Gf/Gv* were non-negligibly related to academic achievement, the best predictors of reading and math, respectively, at T2 were reading and math, respectively, at T1.

The third model (M3) Watkins et al. [69] examined fit the data almost indistinguishably from M2. The major difference between the two models was that in M3 reading and math at T1 were specified to be direct causes of *Gc* and *Gf/Gv* at T2. The *Gc*_T1_-reading_T2_ and *Gf/Gv*_T1_-math_T2_ relations for M2 were statistically the same (up to the sign) as the reading_T1_-*Gc*_T2_ and math_T1_-*Gf/Gv*_T2_ relations in M3.

We are not necessarily advocating for a position that academic achievement attributes “cause” intelligence attributes. What we are arguing is that determining the nature of causality between intelligence and academic achievement attributes is much more difficult than showing IQ test scores and standardized academic achievement scores are correlated [70].

Third, correlations provide information about what is likely true in aggregate for a population, not what is true of each and every member in that population [62]. That is, by themselves, correlations provide no information about individuals within that population. Thus, it could be that, in aggregate, intelligence attributes are correlated with academic achievement, but the relation disappears when investigating specific individuals [71]. To show that attributes being assessed by IQ tests have a causal relation to SLD would require showing that a relation exists at the individual level, not just that there is a relation between IQ test scores and academic achievement test scores in the aggregate [72].

If intelligence attributes were causally related to academic difficulties, then we would expect that IQ test scores and academic achievement test scores would be related. The presence of correlations between IQ test scores and academic achievement test scores, however, does not imply that intelligence attributes are causally related to academic difficulties—no matter how robust the correlations. Consequently, assuming that correlations between IQ test scores and academic achievement test scores in a population implies that the former is causally-related to SLD in specific individuals is a false cause fallacy.

#### 3.2.5. Studies That Answer the Wrong Question

The fifth type of support comes from the few experiments designed to study DD/C that ask and answer the wrong questions. As described in Section 3.1, support for a SLD identification method needs to come from studies that answer questions about diagnostic utility. Instead, experimental studies that are cited as supporting DD/C answer other questions (e.g., mean differences between groups). Feifer et al. [73] study provides a typical example.

Feifer et al. [73] divided 283 elementary school students into five reading SLD groups (*n*s ranging from 29–42) based on the DD/C or other “conceptually similar” PSW approaches, as well as a comparison group of students without identified SLD (n=35). Each participant was given 14 IQ test subtests and three academic achievement subtests from nationally-normed instruments producing norm-referenced scores. Feifer et al. then regressed each academic achievement score on the 14 IQ test scores in each of the six groups, separately, and subsequently compared what IQ test scores had a “statistically significant” relation with academic achievement scores as well as compared mean differences. Because the IQ subtests were differently predictive across the groups, Feifer et al. concluded that “different cognitive profiles emerge with respect to processing strengths and weaknesses” (p. 26) for the different types of SLD in reading.

We ignore the fact that Feifer et al. [73] procedures violated well-known guidelines for regression analysis, and instead focus on the fact that they answered the wrong question about DD/C.^12^ They answered the question: are there mean differences in IQ subtest scores between SLD groups formed using DD/C (or “conceptually similar” PSW approaches)? What they should have asked is: can differences in IQ subtest scores scores accurately classify an SLD in reading? If so, can they do so any better than using academic achievement measures alone?

The assumption that research answering the wrong questions supports the DD/C method is a form of the strawman fallacy.

## 4. Problems Associated with the DD/C Method

### 4.1. Untenable Assumptions

All discrepancy frameworks, including DD/C, have some major assumptions that are untenable based on the current state of knowledge about intelligence and its measurement.

#### 4.1.1. Measurement

The assumptions related to measurement are:

1. If scores from nationally-normed and standardized IQ and achievement tests are made to have the same arbitrary mean and SD values, then scores from these tests are directly comparable.
2. If the scores from nationally-normed IQ and achievement tests are standardized and put on a norm-referenced scale, then those scores have interval measurement properties.
3. Because of assumptions 1 and 2, the observed differences in norm-referenced scores between nationally-normed IQ and achievement tests map directly onto the differences in the attributes represented by the test scores.

Scores from IQ and academic achievement tests are, *at best*, on ordinal scales [75,76]. One major difference between attributes measured on an ordinal scale versus those measured on an interval scale is *additivity* [77]. When a measurement scale has additivity, it means that, across *all* possible values, any differences of the same magnitude represent the same difference in the attribute the scales’ numerals represent. Specifically, additivity is present for scale when the following is true for its values (*V*):

- If $V_{c}-V_{b}=V_{z}-V_{y}$ and $V_{b}-V_{a}=V_{y}-V_{x}$ (for any ordered series where c>b>a and z>y>x),
- then $V_{c}-V_{a}=V_{z}-V_{x}$ [78].

**Figure 1:**
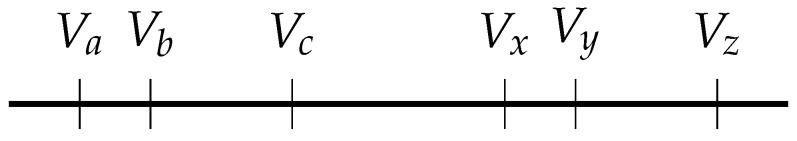
Geometric representation of additivity.

Using Figure 1, it is relatively easy to see that if *V* represents values for the attribute of length, then length scales have additivity. As an example, let $V_{a}$, $V_{b}$, and $V_{c}$ be 65 cm, 70 cm and 80 cm, respectively, and let $V_{x}$, $V_{y}$, and $V_{z}$ be 100 cm, 105 cm and 115 cm, respectively. Then, 80 cm−70 cm=115 cm−105 cm because the differences represent the same amount of length: 10 cm. Likewise, 70 cm−65 cm=105 cm−100 cm because these differences represent the same amount of length: 5 cm. Consequently, if the length scale is additive, then 80 cm−65 cm should represent the same amount of length as 115 cm−100 cm—which is verifiably true. Thus, length scales have additivity.

What happens if *V* represents, say, values for the attribute of Comprehension-Knowledge (*Gc*)? Do scores on an IQ scale have additivity? (For more information on the IQ scale, see Section 4.1.2.)^13^ Let $V_{a}$, $V_{b}$, and $V_{c}$ be 65 IQ, 70 IQ and 80 IQ, respectively, and let $V_{x}$, $V_{y}$, and $V_{z}$ be 100 IQ, 105 IQ and 115 IQ, respectively. Assume we could demonstrate that an individual with 80 IQ and an individual with 70 IQ differ in *Gc* as much as individuals with 115 IQ and 105 IQ. Likewise, assume we could demonstrate that individuals with 70 IQ and 65 IQ differ in *Gc* the same amount as individuals with 105 IQ and 100 IQ. Does it then follow that difference in *Gc* between the individuals with 80 IQ and 65 IQ is the same amount of difference in *Gc* as the individuals with 115 IQ and 100 IQ? No [79].

Because values on the IQ scale represents the number of SDs an individual’s raw score is from some reference point (e.g., the mean/median score from the norming group) and raw scores are made to follow some type of symmetrical distribution (e.g., normal), it would be very difficult to make the argument that a change from 65 IQ to 80 IQ represents the same level of increase in *Gc* as a change from 100 IQ to 115 IQ. First, value differences closer to the mean/median represent less meaningful change in the attribute than value differences further away from the mean/median [80]. Second, and most problematic, is that the SD is not a measurement unit.

A unit is “real scalar quantity, defined and adopted by convention, with which any other quantity of the same kind [of attribute] can be compared to express the ratio of the two quantities as a number” [81] (p. 6). In other words, it is a particular example of the attribute that is used as a reference. For example, the base unit for (thermodynamic) temperature is the Kelvin, defined as “the fraction 1/273.16 of the thermodynamic temperature of the triple point of water” [82] (p. 114).^14^ Compare this to the definition of a SD, “a common measure of the scatter or dispersion of a set of measurements, equal to the square root of the mean of the squares of the deviations” [83] (definition 2.d). Knowing that individual’s *Gc* is, say, 100 IQ just indicates that the individual’s raw scores on the instruments used to assess *Gc* were the same as the average raw scores in the norming group. It provides no information about the individual’s actual level of *Gc*.

The major implication of IQ and academic achievement tests using ordinal measurement scales is that the values have heterogeneous orders [84]. That is, 80 IQ−65 IQ likely represents an amount of *Gc* that is different than 115 IQ−100 IQ. Thus, the values’ meaning has to be tied to some reference (e.g., norming sample) instead of a unit.

Historically, most SLD research—like much other research in psychology—has been predicated on the ideas that psychological attributes are being measured on interval scales [85]. This assumption likely has relatively minor consequences for many aggregate-type statistics (e.g., correlation) because parametric and nonparametric versions are often quite similar [86]. It becomes much more problematic when examining score differences within an individual—the foundation of all IAD and PSW methods—and when inferring deficits from norm-referenced scores alone [87] (chapter 3).

#### 4.1.2. Score Precision

The DD/C method requires that all test scores be placed on an IQ scale (mean: 100, SD: 15) so they can be directly compared. Thus, if the original scores were on, e.g., Scaled Score scale (mean: 10, SD: 3), they have to be transformed to the IQ scale. This is done through the linear transformation in Equation (1):

(1) $$S_{N}=M_{N}+SD_{N}\times \frac{S_{O}-M_{O}}{SD_{O}},$$

where the *O* subscript represents the original score scale, *N* represents the new score scale, *S* is an individual’s score, *M* is the sample mean, and SD is the sample standard deviation.

From measurement theory, we know that linear transformations (e.g., Equation (1)) are allowable for numerals on both interval and ordinal measurement scales [87]. The differences between the applications is that interval scale values are quantitative, so there is an infinite number of values between any two numbers. Thus, there is no loss or gain of information from going from one scale to another (i.e., g to kg). The scale chosen is the one that is most convenient for a given application.

When the transformation in Equation (1) is applied to ordinal scale values, it preserves their rank orders. Any score interpretations related to one score being ≥ to another score do not change. Interpretations involving score differences, however, can change because it can imply there is more or less information in the scores than there actually are.

Take values on the Scaled Score scale (ScS), for example. It has an SD of three, meaning that each value represents one-third of a SD. If these scores are transformed to the IQ scale, however, now each value represents one-fifteenth of a SD. In other words, the scores now give the appearance of having more information because each IQ scale score value ostensibly represents a more precise level of the attribute.

A more concrete example is given in Table 3. A score of 8 ScS has percentile rank (PR) values ranging from 21 PR to 30 PR. Only at 31 PR does the value change to 9 ScS. Values on the IQ scale, however, do not follow the same pattern. Values of 21 PR to 30 PR are equivalent to 88 IQ to 92 IQ, respectively. Thus, a value of 8 ScS is not only equivalent to 88 IQ, but all the scores between 88 IQ and 92 IQ (i.e., 89 IQ, 90 IQ and 91 IQ).

In other words, information that was not available in the original Scaled Score scale now appears to be present on the IQ scale. How can this be, though? There is nothing about a score transformations that provides additional information about an attribute. However, that is exactly what appears to happen as a consequence of these score transformations. Thus, these transformations give the appearance of false score precision—a precision that does not exist.

One could argue that putting all scores on the same scale is convenient, and any ensuing false precision that results is a minimal price to pay for the convenience. That argument may be plausible if no high-stakes decisions were associated with the scores, but that is not the case with SLD diagnoses. According to the DD/C criteria, a score <90 IQ is needed to have a normative weakness/deficit. Thus, the same Scaled Score value (i.e., 8) can concurrently represent IQ scale scores that are both in the average range (i.e., not identified as SLD) and a normative weaknesses/deficit (i.e., possibly identified as SLD).

Admittedly, the DD/C developers do not always make hard distinctions between score values. For example, they wrote “sole reliance on standard scores and cut points should be avoided in any diagnostic approach to determining disability, particularly SLD” [38] (p. 124). In the same source [38] (pp. 229, 237, 242–245, 268, 279) and in a subsequent publication [17] (pp. 338, 359–361, 406), however, they spend multiples pages discussing the importance of scores being ≥90 IQ and differentiating those between 85 IQ to 90 IQ versus those <85 IQ. Thus, readers are left to wonder about the importance of the criteria they provide for scores to be included/excluded in these categories. If an examinee took a reading test and earned a score of 90 IQ, would that be sufficient evidence for Level 1? What about 91 IQ? 92 IQ or 93 IQ?

We suspect that DD/C developers’ answer to this question would be something along the lines of: it depends on the evaluator’s clinical judgment, e.g., [17] (p. 406). In fact, Flanagan et al. [17] (p. 409) even provided an example of “Amanda” who had a math fluency score of 89 IQ that they considered to be a strength based on their own clinical judgment. If the DD/C method is designed to be an approach where clinical judgment is heavily emphasized and the inclusion/exclusion criteria for average performance versus weakness/deficit are only suggestions, then the method should be reclassified as *exploratory*, with the goals of hypothesis generation and pattern detection [88]. The price for being an exploratory method is that hypothesis confirmation (e.g., SLD identification) cannot be an outcome. Instead, DD/C, as an exploratory process, would develop hypotheses about why a student is having an academic difficulty; however, actually making an SLD identification decision would require a subsequent confirmatory process using separate data and analyses [89].

Our understanding of the DD/C process is that the developers used an exploratory philosophy, but believe the resulting information allows for confirmatory conclusions. For example, Flanagan et al. [38] stated their philosophy about psychological assessment as one that is consistent with an exploratory method: “We have long been advocates of “intelligent” testing—the notion that a profile of test scores is meaningless unless it is brought to life by the clinical observations and astute detective work of knowledgeable examiners” (p. xiii). However, at the same time, they argued that the process is a confirmatory method because individuals who meet criteria for all five DD/C levels can be identified as having a SLD. Combining the two types of methods into a single processes using the same data and analyses will likely result in decisions heavily influenced by cognitive biases [90]—something that has plagued many other informal/subjective/impressionistic methods in psychology that rely on clinical judgement [91].

#### 4.1.3. Score Exchangeability

Scores are exchangeable when they measure the same attribute and are expressed in the exact same unit [92]. Conceptually, the exchangeability assumption is not tenable with most intelligence tests because there are no uniform definitions/operationalizations of the attributes by which scholars agree to use when developing instruments [68,93,94]. Granted, there has been a lot of work in the last few decades in developing uniform definitions of these attributes [95]. Nonetheless, just because two (sub)tests use the same or similar names for the attributes, they purport to assess does not mean they are equivalent and the tests’ scores are exchangeable. For example, the way one test assesses “processing speed” can look very different from that of another test [96].

Flanagan et al. [38] argued that test scores representing CHC broad abilities are exchangeable because they are correlated with each other.^15^ This is another instance of asking the wrong question. If scores are exchangeable, they will have a strong positive correlation, but, the converse is not true. Just because scores have a strong positive correlation does not mean that they are exchangeable. Height and weight is an example—the correlation between the attributes is typically high, yet who would argue that 100 kg is the same as 100 m?

Floyd et al. [99] examined scores ostensibly assessing the same CHC broad ability attributes across multiple IQ tests and found the scores were not exchangeable. Miciak et al. [100] found similar results for nationally-normed achievement tests. Thus, when using the DD/C method, SLD identification results can differ substantially depending on the tests used to assess the attributes.

#### 4.1.4. Functional

Functionally, a major assumption of all discrepancy frameworks—both IAD and PSW—is that individuals who have academic difficulties and meet the discrepancy criteria are qualitatively different from those individuals who have academic difficulties but do not meet the discrepancy criteria. That is, students who meet diagnostic criteria for SLD using IQ test scores have poor academic achievement for *different* reasons than students who do not meet the diagnostic criteria [19].

Two meta-analyses have been published summarizing research as it relates to this assumption for SLD in reading [27,101]. Both concluded that—for most intelligence attributes assessed—especially those closely related to the reading disability—there was considerable overlap between the two groups. In other words, IQ test scores representing the core intelligence attributes that are most closely related to reading disabilities do not significantly discriminate students with and without a SLD in reading. Both meta-analyses used studies from IAD-based identification criteria instead of PSW. While we believe the results from the IAD studies are applicable to PSW since both methods have the same assumption, this is something that needs to be verified empirically. We are not aware of any published study that has examined this using the PSW method.

A related assumption is that certain IQ and achievement test score patterns only, or mostly, occur in the presence of SLD. Otherwise, these PSW would not be a marker for SLD. Little research has been done investigating this assumption, but what has been done indicates that it is not uncommon to exhibit a PSW in IQ and academic achievement test scores. For example, using a nationally representative sample of over 7000 individuals who were given the same IQ and achievement tests, Stafford [102] found that 42% met the Level 3 DD/C criteria and 37% met the Level 1 and Level 3 criteria. Thus, either SLD prevalence is grossly underreported or PSW are quite common; if the latter, then their status as a SLD marker is questionable.

### 4.2. Experiments Not Supporting the Dual Discrepancy/Consistency Method

The DD/C method has been around for over a decade in one form or another. However, there are only a few published experimental studies of it. The experimental studies that have been published—especially by individuals without CoIs—have shown problems with the method. Specifically, it lacks sensitivity in identifying individuals with an SLD.

Miciak et al. [103] examined the DD/C method in students who had previously demonstrated an inadequate response to supplementary reading instruction (i.e., RtI Tier 2 intervention). They found only 17% to 25% of the students met criteria for a SLD in some area of reading using thresholds of 85 IQ and 90 IQ, respectively. In addition, the difference in scores on academic achievement tests between students who did and did not meet the DD/C criteria, using either threshold, was small. A pattern that did emerge was that decreasing the threshold score for meeting DD/C Level 1 criteria (i.e., a weaknesses/deficits in an area of academic achievement) resulted in groups of students with more severe difficulties in other areas of academic achievement. That is, difficulty in achievement was better predicted by other tests of achievement than patterns of IQ test scores.

Stuebing et al. [36] simulated data to investigate DD/C. Results indicated that only a small proportion of the observation met SLD identification criteria using the DD/C method. The DD/C method demonstrated high specificity and high NPV, but this was expected due to the low base rate of SLD. The DD/C method displayed low sensitivity and low PPV, suggesting that even if “true” PSWs exist and are meaningful, classifications based on typical IQ and achievement test scores will not reliably identify those individuals. Not only was the accuracy of identifying those with true SLD relatively low, but also about half of the individuals identified with SLD were considered to be false positives.

Kranzler et al. [104] examined diagnostic utility of the DD/C using data from a nationally co-normed IQ and achievement tests. The DD/C method identified a low number of children as having a SLD. The method was reliable in detecting true negatives (i.e., those not having a SLD) and demonstrated high NPV. Again, this was expected given the low base rate for SLD. The PPV and sensitivity were low, however, meaning it was not able to identify those who did have a SLD. This suggests that many students who demonstrate academic deficits will infrequently demonstrate a IQ test score profile that would be identified using DD/C criteria. Their study also raises questions about the extent to which low academic achievement is caused by intra-individual differences in intelligence attributes.

Flanagan and Schneider [42] raised a number of objections to the Kranzler et al. [104] study, one of which was that Kranzler et al. [104] applied DD/C criteria incorrectly. Admittedly, the aim of Kranzler et al. [104] study was not to diagnose SLD, but to “use classification agreement analysis to examine the predicted concordance between cognitive abilities of children and adolescents with and without academic weaknesses within an otherwise normal cognitive profile” [56] (p. 152). Nonetheless, they did base their classifications on analyses of scores using DD/C criteria at Levels 1, 3, and 4 as implemented in the X-BASS software’s precursor program. Thus, the study does provide information pertinent to evaluating the utility of the DD/C method, albeit somewhat limited.

Another criticism Flanagan and Schneider [42] raised was that individual subtests were used as markers for broad abilities. They wrote that subtests do not adequately represent the full range of CHC broad abilities, so it is possible that more favorable diagnostic efficiency statistics would have been obtained if composite scores (i.e., scores comprised of two or more subtests) were used instead. Kranzler et al. [105] and Miciak et al. [106] experimentally examined this assertion. Kranzler et al. [105] re-conducted their original study using composite scores. They found minimal differences and, overall, their results replicated their previous study. Miciak et al. [106] conducted a simulation study comparing the classification accuracy of various PSW permutations using single and multiple indicators. Use of multiple indicators resulted in modest improvements in overall accuracy beyond single indicators. As a result, they concluded that “Finite resources would be better spent directly assessing key academic skills and providing targeted interventions for students in need of assistance rather than administering more cognitive tests” (p. 32).

### 4.3. Cross-Battery Assessment Software System Software

The DD/C method requires using the X-BASS software [107,108]. The X-BASS uses elements of previously released software programs associated with XBA [38] (Appendix H), but it is proprietary. Users input scores from a battery of IQ and achievement tests and specify which scores correspond to required elements of the DD/C model (e.g., scores that represent pertinent intelligence and academic attributes). If the score specified as the intelligence attribute weakness exceeds the critical value derived from the program’s algorithm, it is considered to demarcate a “statistically significant” and “rare” weakness.

The X-BASS software ostensibly provides users with a rigorous method for determining strengths and weaknesses associated with the DD/C model, but it has never undergone peer review so little is known about it. For example, it is unclear exactly how the FCC is calculated. Alfonso [107] noted it was calculated using a “standard formula that incorporates median inter-correlations among and reliabilities of those CHC composites that were judged to be strengths and weaknesses by the evaluator” (p. 53). Does that mean that, say, *Gc* has the same weight in contributing to the FCC regardless of the subtests a clinician used to assess *Gc*? Or do the weights vary by the subtests used? In either case, how does subtest selection influence the predictive ability of the FCC? As another example, Flanagan et al. [17] (pp. 377, 379) wrote that the X-BASS “corrects for false negatives” when determining if there are score discrepancies. However, the correction procedures are not described anywhere, so users are left to wonder: how were these procedures developed and validated?

These are the types of questions that need to be answered to vet the software thoroughly [109]. Until such studies are completed, this aspect of DD/C is likely best considered a “known unknown”, meaning that more information is needed before it can be evaluated rigorously.

### 4.4. Evidence-Based Assessment

Evidence-based assessment (EBA) as an approach to clinical evaluation that uses research and theory to guide the assessment process [110]. Specifically, EBA involves (a) an evaluation of the accuracy and usefulness of this complex decision-making task in light of potential errors and biases in data synthesis and interpretation, (b) the costs associated with the assessment process and, (c) the impact the assessment had on clinical outcomes for the individuals being assessed.

Based on the evidence presented in this article, the DD/C method does not appear to aid clinicians in making accurate decisions about SLD. Specifically, the criteria appear to be problematic because they are not sensitive to distinguishing between those who have low achievement but not SLD versus those with a low achievement test score and SLD. Thus, the DD/C method does not appear to meet the first criterion for EBA.

Since the DD/C method requires gathering multiple scores from IQ tests—in addition to the myriad of other data required to make decisions related to exclusionary factors—using DD/C increases the time and financial costs associated with the SLD identification process [111,112]. For example, assessing all seven CHC broad ability using two subtests—what Flanagan et al. [38] recommend—requires administering, scoring, and interpreting *at least* 14 subtests, which can take many hours. Moreover, if the two subtests that assess the same broad ability happen to be discrepant, an additional subtest needs to be administered, which adds additional time. Thus, the DD/C does not appear to meet the second criterion for EBA.

The third criterion for EBA—the impact the assessment has on clinical outcomes for the individuals being assessed—is essentially a question of treatment utility. That is, do the score patterns from IQ tests aid in developing academic interventions [113]? There have been two meta-analyses examining the treatment utility of scores derived from IQ tests [114,115]. Both found that differences in IQ test score patterns had a much weaker relation to improvement during intervention than baseline academic achievement levels. This is consistent with other literature showing that accounting for inter-individual differences in intelligence attributes when implementing academic interventions does not make the interventions more effective [116,117]. Thus, the DD/C method does not appear to meet the third criterion for EBA.

Consequently, since DD/C does not meet any of the criteria for EBA, we believe that it cannot currently be classified as an evidence-based method for identifying SLD. Consequently, DD/C likely does not qualify at this time as an “other alternative research-based procedure” for identifying SLD via IDEA. Although the criteria for a SLD identification procedure being “research-based” are not specified in the IDEA regulations, it is difficult to imagine any set of scientific criteria under which DD/C would currently be found acceptable given: (a) its untenable assumptions, (b) the lack of experimental studies supporting its use, and (c) the experimental literature not supporting its use.

## 5. Conclusions

In this article, we described the DD/C method for identifying an SLD. Although PSW methods in general, and DD/C methods in particular, are often described by their proponents as something new and revolutionary, this is not the case. These methods have been articulated in the professional literature for decades and prominent assessment scholars regard them as a reissuing of cognitive profile analysis that have long been regarded as problematic [118]. In spite of the growing fascination with the use of this method and its related assessment products (e.g., X-BASS), we have identified and elaborated on several conceptual and psychometric shortcomings associated with the method.

Although our understanding of these matters remains is far from certain, almost every study that has directly assessed the potential diagnostic utility of the DD/C method has furnished evidence that it is not very useful for identifying individuals with SLD. Nevertheless, the method continues to be disseminated widely in questionable publications [119] and professional development workshops/seminars by individuals with CoIs. Thus, experimental outcomes are rarely disclosed to consumers. When they are discussed, the results of these investigations are almost always countered with appeals to clinical wisdom. Lilienfeld et al. [120] regarded this notion as the *alchemist’s fantasy*—where advocates seem to believe that the powers of clinical intuition enable them to transform questionable practices into clinical gold. We were unable to locate any compelling research evidence linking use of the DD/C method with increased diagnostic precision or developing/implementing viable positive educational outcomes—a situation even PSW proponents now acknowledge [121]. Instead, existing evidence indicate that clinicians employing the DD/C method “will spend a great deal of time conducting assessments that have a very low probability of accurately identifying true SLD” [56] (p. 134).

The Oxford English Dictionary defines *misuse* as “wrong or improper use; misapplication; an instance of this” [122]. Based on the lack of evidence currently available showing the DD/C method has utility in identifying students with SLD in conjunction with the extant evidence showing problems with the DD/C method, we believe continued use of the DD/C method to identify individuals with SLD represents a misuse of IQ scores. Although IQ scores have been controversial since their development, and there is still great debate about the attributes the scores represent, there is little doubt that they have utility for multiple “real world” situations [123,124]. Using them to identify SLD, however, does not appear to be one of them.

## Figures and Tables

**Table 1:** Attributes related to reading according to DD/C and sample tests used to assess reading.

Cattell–Horn–Carroll		Reading Subtests ^2^
Broad Ability ^1^	Narrow Ability ^1^	
*Gc*	Language development (LD)	Reading Comprehension
*Gc*	Lexical knowledge (VL)	Reading Vocabulary
*Gc*	Listening ability (LS)	Decoding Fluency
*Ga*	Phonetic coding (PC)	Phonological Processing
*Glr*	Naming facility (NA)	Word Recognition Fluency
*Glr*	Associative memory (MA)	Nonsense Word Decoding
*Gwm*	Memory span (MS)	Letter and Word Recognition
*Gwm*	Working memory capacity (WM)	Silent Reading Fluency
*Gs*	Perceptual Speed (P)	

^1^ Taken from Flanagan et al. [17] (pp. 352–353). ^2^ Taken from Flanagan et al. [17] (p. 340), under the *KTEA-3* column.

**Table 2:** Example of a classification table needed for utility information.

D/DC Method Decision	True Status	
	Positive (SLD)	Negative (No SLD)
Positive (SLD)	True Positive	False Positive
Negative (no SLD)	False Negative	True Negative

**Table 3:** Score conversion table.

Percentile	Scaled Score Scale	IQ Scale
31	9	93
30	8	92
27	8	91
26	8	90
25	8	90
24	8	89
23	8	89
22	8	88
21	8	88
20	7	87

*Note*. Values within the dashed lines are associated with the Scaled Score value of 8. Scaled Score and IQ scores were calculated using the quantile function of a normal distribution and rounding to the nearest integer. For example, the Scale Score and IQ scale values, respectively, for the 31th percentile were calculated as $10+3\Phi^{-1}\left(0.31\right)$ and $100+15\Phi^{-1}\left(0.31\right)$, where $\Phi^{-1}$ is the probit function.

## Footnotes

1. We use the term *intelligence* throughout this article to refer to the class of attributes within the general domain of cognitive ability, not any particular attribute within that domain. We use the term *IQ test* to refer to any instrument designed to assess intelligence attributes.

2. We use the term *cognitive profile* broadly to refer to intra-individual score patterns on one or more IQ tests. The patterns could be created directly from the norm-referenced scores or may be ipsatized (i.e., subtract each score from the average of the scores). Likewise, the patterns could be created from subtest scores or composite scores comprised of values from a subset of the subsets (e.g., index scores on the Wechsler scales).

3. We use Cattell’s [7] definition of *experiment* as a “recording of observations, quantitative or qualitative, made by defined and recorded operations and in defined conditions, followed by examination of the data, by appropriate statistical and mathematical rules” (p. 22). These studies may, or may not, involve variable manipulation.

4. Flanagan et al. [41] developed the method in 2002 as part of their guidelines for using XBA to identify SLD. Since then, they have not been consistent with their use of terms. Historically, it has been referred to as the “Operational Definition of SLD”, “CHC-based operational definition of SLD”, or has been used interchangeably with XBA. The term *dual discrepancy/consistency* (DD/C) was introduced in 2013, but there have still been inconsistencies in term usage. For example, Flanagan and colleagues named their 2013 test-analysis software *Cross-Battery Pattern of Strengths and Weaknesses Analyzer* and named the latest iteration of this software the *Cross-Battery Assessment Software System*. Flanagan and Schneider [42] requested that the term *dual discrepancy/consistency* be used, so we use it throughout this article.

5. In their most recent publication, Flanagan et al. [17] (p. 401) wrote that the DD/C method now requires that clinicians categorize *all* IQ and achievement test scores as either a “strength” or “weakness”.

6. Flanagan and colleagues are not consistent in their terms or definitions. We use the terms and definitions they provided in their most recent publication [17], which differ somewhat from previous publications [38] (pp. 242 & 244 (Table 4.3)). Moreover, they have also stated in DD/C workshops that clinicians can use whatever criteria they deem important for documenting a weakness [43].

7. Elsewhere, Flanagan and Alfonso [44] have said that, for a reading SLD, the primary weakness cannot be *Gc* due to the overlap with achievement. The inconsistency in what can and cannot count as a primary weakness in intelligent attributes is problematic but is not the particular focus of this article.

8. Clinicians can opt to use the Alternative Cognitive Composite (ACC) instead of the FCC. The ACC is “any cognitive composite derived from an intelligence or cognitive ability battery that is a good estimate of overall cognitive ability and, in the evaluator’s judgment, is a better estimate than the FCC” [17] (p. 413). Thus, the determination of weaknesses in attributes related to intelligence and academic achievement can be idiosyncratic to each clinician.

9. This table could be generalized to the situation with >2 true statuses (e.g., dimensional models of SLD) as well as >2 outcomes (e.g., method produces an “uncertain” diagnosis) [51].

10. There is not currently a “gold-standard” method for identifying SLD, so there is no way to establish universally someone’s *true status* in Table 2 when using data collected from actual individuals. Instead, it is usually defined on a study-by-study basis. With simulation studies, this is much less of a problem since individuals’ *true status* is part of the parameters specified in the simulation process [53].

11. We state the DD/C developers profit because in order for clinicians to use DD/C method, they have to acquire DD/C books, attend DD/C workshops, or get “certified” in XBA, in addition to purchasing a license for the X-BASS program. Profiting off of clinicians’ choices is not unique to the DD/C developers, nor is it necessarily an unethical practice.

12. Some examples of major problems with their analysis are running regressions on samples of very small size, not assessing if they violated the assumptions inherent in regression, and completing regressions for each group separately [74].

13. We acknowledge that is confusing to have an IQ scale that exists independent of IQ tests. We attempt to maximize clarity by using the term *IQ scale* whenever we are referring to scores from an instrument assessing some arbitrary attribute, and the term *IQ test* when referring to an instrument assessing some intelligence attribute.

14. The triple point of a substance is the temperature and pressure at which the its gas, liquid, and solid phases coexist in equilibrium.

15. Flanagan et al. [38] (p. 399) wrote that in developing the XBA guidelines—which are used in the DD/C method—they used results from factor analytic studies for their subtest classifications. Factor analysis is just a method of reducing the information in a set of correlations, however. The use of factor analysis cannot substitute for the lack of conceptual equivalence or strong theory about how why scores are related [97,98].

## Abbreviations

DD/C	Dual discrepancy/consistency
PSW	Pattern of strengths and weaknesses
SLD	Specific learning disability
XBA	Cross-battery assessment

## Appendix A

For convenience, Table 2 is shown again as Table A1.

**Table A1:** Example of a classification table needed for utility information.

D/DC Method Decision	True Status	
	Positive (SLD)	Negative (No SLD)
Positive (SLD)	True Positive ( Hits)	False Positive ( False Alarms)
Negative (no SLD)	False Negative ( Misses)	True Negative ( Correct Rejections)

The base rate is calculated via

(A1) $$\mathrm{Base}\;\mathrm{Rate}=\frac{\mathrm{Hits}+\mathrm{Misses}}{\mathrm{All}\;\mathrm{Individuals}},$$

and is interpreted as the prevalence of SLD in a population.

Specificity is calculated via

(A2) $$\mathrm{Specificity}=\frac{\mathrm{Correct}\;\mathrm{Rejections}}{\mathrm{Correct}\;\mathrm{Rejections}+\mathrm{False}\;\mathrm{Alarms}},$$

and is interpreted as the probability an individual without SLD will be identified as such using DD/C criteria.

Sensitivity is calculated via

(A3) $$\mathrm{Sensitivity}=\frac{\mathrm{Hits}}{\mathrm{Hits}+\mathrm{Misses}},$$

and is interpreted as the probability an individual with SLD will be identified as such using DD/C criteria.

Negative predictive value (NPV) is calculated via

(A4) $$\begin{array}{c} \begin{array}{cc} \mathrm{NPV} & =\frac{\mathrm{Correct}\;\mathrm{Rejections}}{\mathrm{Misses}+\mathrm{Correct}\;\mathrm{Rejections}}\\ & =\frac{\mathrm{Base}\;\mathrm{Rate}\times \mathrm{Sensitivity}}{\mathrm{Base}\;\mathrm{Rate}\times \mathrm{Sensitivity}+(1-\mathrm{Base}\;\mathrm{Rate})\times (1-\mathrm{Specificity})}, \end{array} \end{array}$$

and is interpreted as the probability an individual not identified as having a SLD using DD/C criteria actually does not have a SLD.

Positive predictive value (PPV) is calculated via

(A5) $$\begin{array}{c} \begin{array}{cc} \mathrm{PPV} & =\frac{\mathrm{Hits}}{\mathrm{Hits}+\mathrm{False}\;\mathrm{Alarms}}\\ & =\frac{(1-\mathrm{Base}\;\mathrm{Rate})\times \mathrm{Specificity}}{\left(1-\mathrm{Base}\;\mathrm{Rate})\times \mathrm{Specificity}+\mathrm{Base}\;\mathrm{Rate}\times (1-\mathrm{Sensitivity}\right)}, \end{array} \end{array}$$

and is interpreted as the probability an individual identified as having a SLD using DD/C criteria actually has a SLD.
